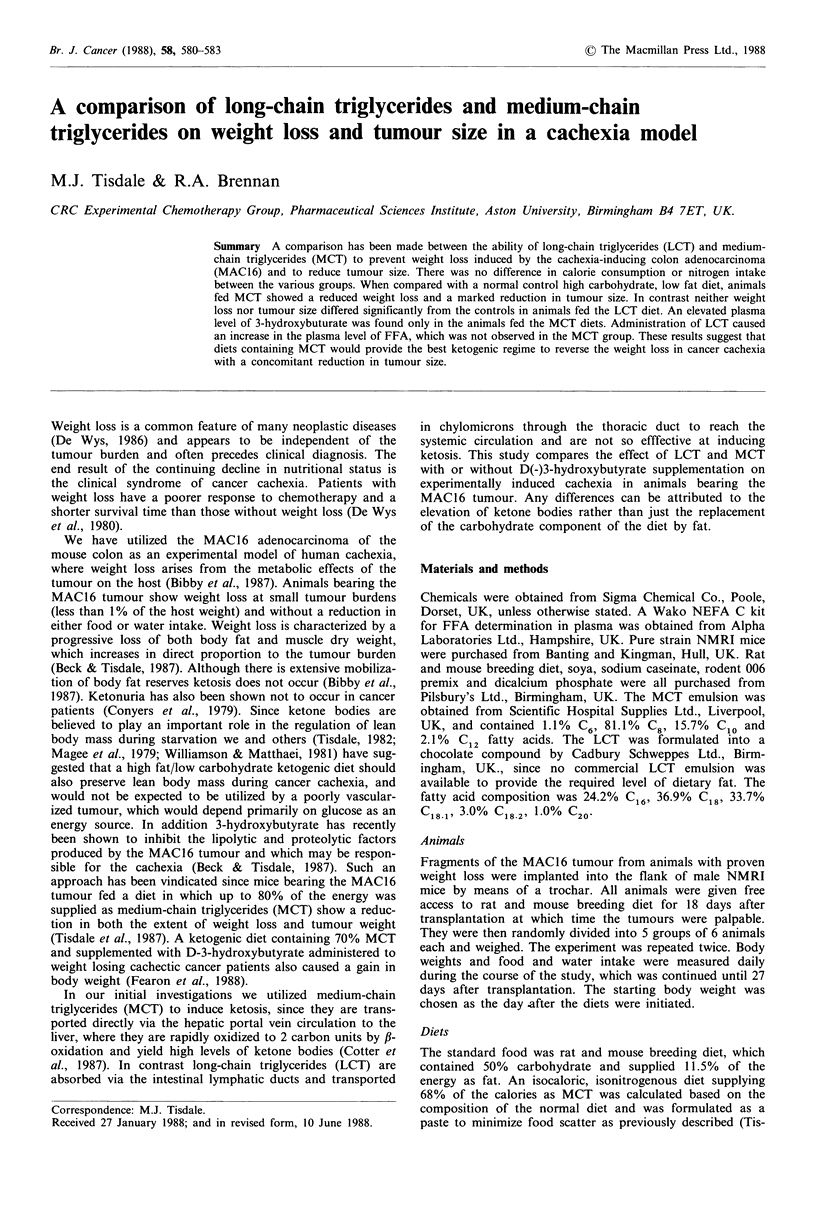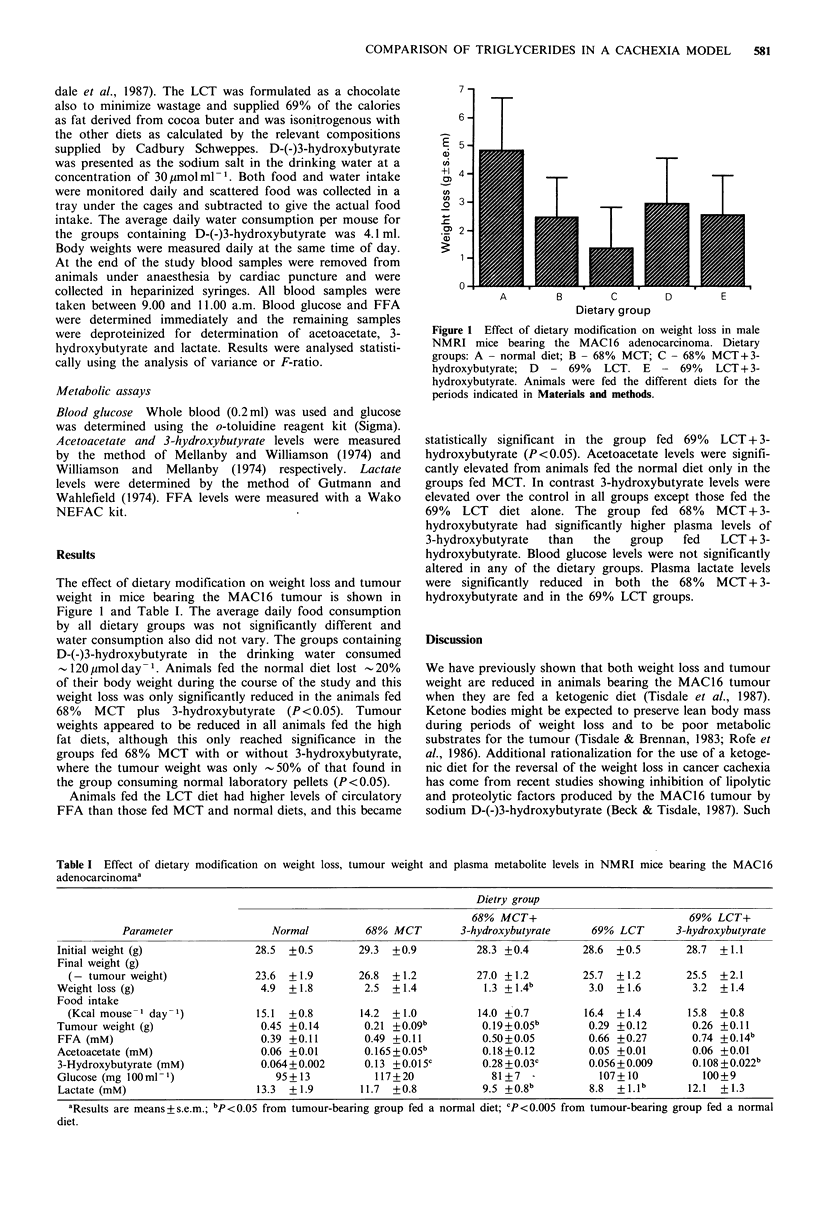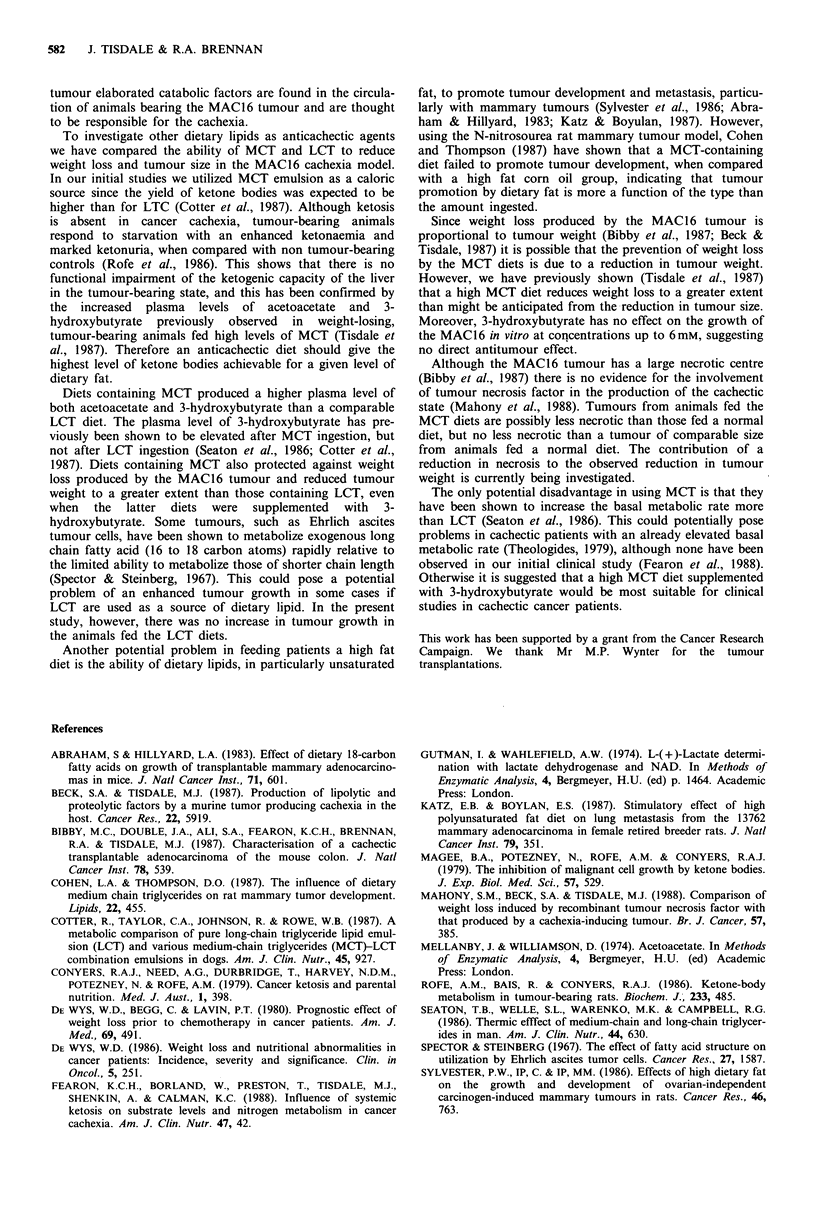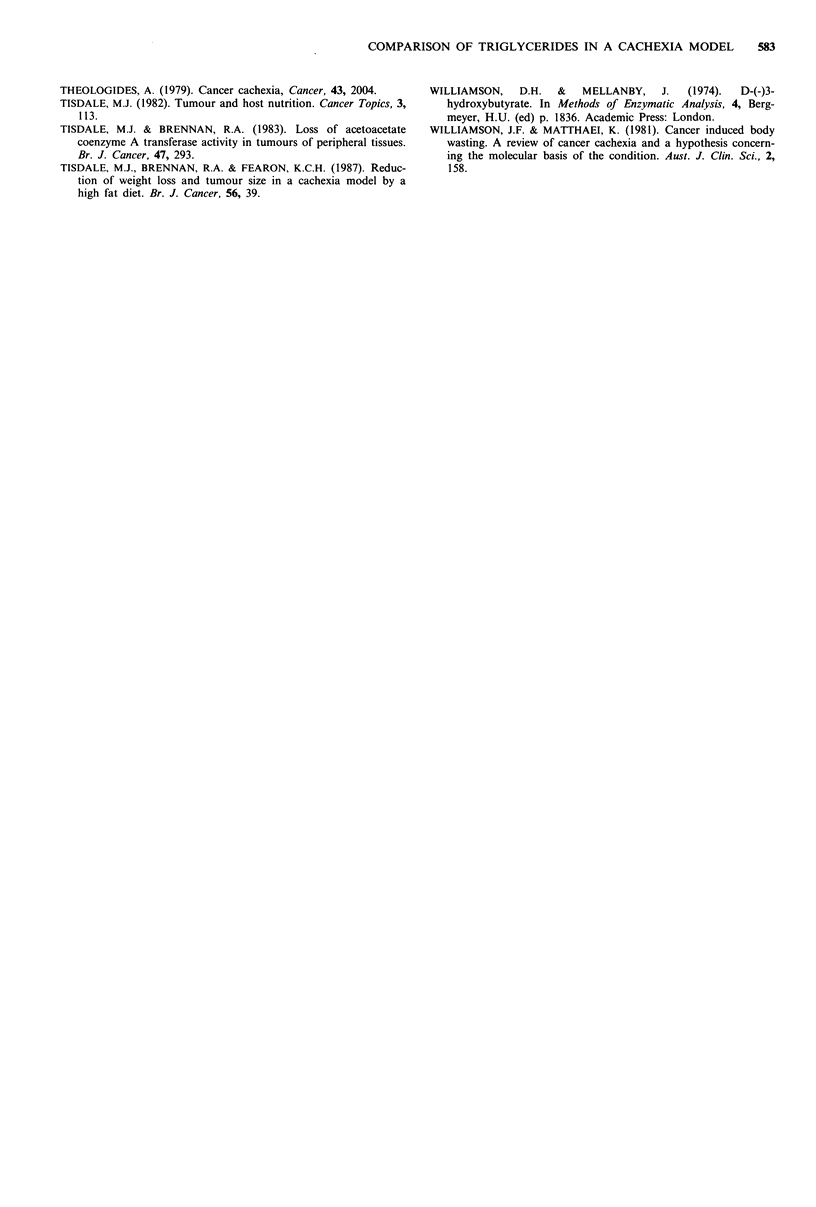# A comparison of long-chain triglycerides and medium-chain triglycerides on weight loss and tumour size in a cachexia model.

**DOI:** 10.1038/bjc.1988.263

**Published:** 1988-11

**Authors:** M. J. Tisdale, R. A. Brennan

**Affiliations:** Pharmaceutical Sciences Institute, Aston University, Birmingham, UK.

## Abstract

A comparison has been made between the ability of long-chain triglycerides (LCT) and medium-chain triglycerides (MCT) to prevent weight loss induced by the cachexia-inducing colon adenocarcinoma (MAC16) and to reduce tumour size. There was no difference in calorie consumption or nitrogen intake between the various groups. When compared with a normal control high carbohydrate, low fat diet, animals fed MCT showed a reduced weight loss and a marked reduction in tumour size. In contrast neither weight loss nor tumour size differed significantly from the controls in animals fed the LCT diet. An elevated plasma level of 3-hydroxybuturate was found only in the animals fed the MCT diets. Administration of LCT caused an increase in the plasma level of FFA, which was not observed in the MCT group. These results suggest that diets containing MCT would provide the best ketogenic regime to reverse the weight loss in cancer cachexia with a concomitant reduction in tumour size.


*Br. J. Cancer* (1988), **58**, 580–583 © The Macmillan Press Ltd., 1988

### A comparison of long-chain triglycerides and medium-chain triglycerides on weight loss and tumour size in a cachexia model

M.J. Tisdale & R.A. Brennan

*CRC Experimental Chemotherapy Group, Pharmaceutical Sciences Institute, Aston University, Birmingham B4 7ET, UK.*

**Summary** A comparison has been made between the ability of long-chain triglycerides (LCT) and medium-chain triglycerides (MCT) to prevent weight loss induced by the cachexia-inducing colon adenocarcinoma (MAC16) and to reduce tumour size. There was no difference in calorie consumption or nitrogen intake between the various groups. When compared with a normal control high carbohydrate, low fat diet, animals fed MCT showed a reduced weight loss and a marked reduction in tumour size. In contrast neither weight loss nor tumour size differed significantly from the controls in animals fed the LCT diet. An elevated plasma level of 3-hydroxybuturate was found only in the animals fed the MCT diets. Administration of LCT caused an increase in the plasma level of FFA, which was not observed in the MCT group. These results suggest that diets containing MCT would provide the best ketogenic regime to reverse the weight loss in cancer cachexia with a concomitant reduction in tumour size.

Weight loss is a common feature of many neoplastic diseases (De Wys, 1986) and appears to be independent of the tumour burden and often precedes clinical diagnosis. The end result of the continuing decline in nutritional status is the clinical syndrome of cancer cachexia. Patients with weight loss have a poorer response to chemotherapy and a shorter survival time than those without weight loss (De Wys *et al.*, 1980).

We have utilized the MAC16 adenocarcinoma of the mouse colon as an experimental model of human cachexia, where weight loss arises from the metabolic effects of the tumour on the host (Bibby *et al.*, 1987). Animals bearing the MAC16 tumour show weight loss at small tumour burdens (less than 1% of the host weight) and without a reduction in either food or water intake. Weight loss is characterized by a progressive loss of both body fat and muscle dry weight, which increases in direct proportion to the tumour burden (Beck & Tisdale, 1987). Although there is extensive mobilization of body fat reserves ketosis does not occur (Bibby *et al.*, 1987). Ketonuria has also been shown not to occur in cancer patients (Conyers *et al.*, 1979). Since ketone bodies are believed to play an important role in the regulation of lean body mass during starvation we and others (Tisdale, 1982; Magee *et al.*, 1979; Williamson & Matthaei, 1981) have suggested that a high fat/low carbohydrate ketogenic diet should also preserve lean body mass during cancer cachexia, and would not be expected to be utilized by a poorly vascularized tumour, which would depend primarily on glucose as an energy source. In addition 3-hydroxybutyrate has recently been shown to inhibit the lipolytic and proteolytic factors produced by the MAC16 tumour and which may be responsible for the cachexia (Beck & Tisdale, 1987). Such an approach has been vindicated since mice bearing the MAC16 tumour fed a diet in which up to 80% of the energy was supplied as medium-chain triglycerides (MCT) show a reduction in both the extent of weight loss and tumour weight (Tisdale *et al.*, 1987). A ketogenic diet containing 70% MCT and supplemented with D-3-hydroxybutyrate administered to weight losing cachectic cancer patients also caused a gain in body weight (Fearon *et al.*, 1988).

In our initial investigations we utilized medium-chain triglycerides (MCT) to induce ketosis, since they are transported directly via the hepatic portal vein circulation to the liver, where they are rapidly oxidized to 2 carbon units by $\beta$-oxidation and yield high levels of ketone bodies (Cotter *et al.*, 1987). In contrast long-chain triglycerides (LCT) are absorbed via the intestinal lymphatic ducts and transported in chylomicrons through the thoracic duct to reach the systemic circulation and are not so efffective at inducing ketosis. This study compares the effect of LCT and MCT with or without D(-)3-hydroxybutyrate supplementation on experimentally induced cachexia in animals bearing the MAC16 tumour. Any differences can be attributed to the elevation of ketone bodies rather than just the replacement of the carbohydrate component of the diet by fat.

Correspondence: M.J. Tisdale.
Received 27 January 1988; and in revised form, 10 June 1988.

### Materials and methods

Chemicals were obtained from Sigma Chemical Co., Poole, Dorset, UK, unless otherwise stated. A Wako NEFA C kit for FFA determination in plasma was obtained from Alpha Laboratories Ltd., Hampshire, UK. Pure strain NMRI mice were purchased from Banting and Kingman, Hull, UK. Rat and mouse breeding diet, soya, sodium caseinate, rodent 006 premix and dicalcium phosphate were all purchased from Pilsbury's Ltd., Birmingham, UK. The MCT emulsion was obtained from Scientific Hospital Supplies Ltd., Liverpool, UK, and contained 1.1% $C_6$, 81.1% $C_8$, 15.7% $C_{10}$ and 2.1% $C_{12}$ fatty acids. The LCT was formulated into a chocolate compound by Cadbury Schweppes Ltd., Birmingham, UK., since no commercial LCT emulsion was available to provide the required level of dietary fat. The fatty acid composition was 24.2% $C_{16}$, 36.9% $C_{18}$, 33.7% $C_{18.1}$, 3.0% $C_{18.2}$, 1.0% $C_{20}$.

### *Animals*

Fragments of the MAC16 tumour from animals with proven weight loss were implanted into the flank of male NMRI mice by means of a trochar. All animals were given free access to rat and mouse breeding diet for 18 days after transplantation at which time the tumours were palpable. They were then randomly divided into 5 groups of 6 animals each and weighed. The experiment was repeated twice. Body weights and food and water intake were measured daily during the course of the study, which was continued until 27 days after transplantation. The starting body weight was chosen as the day after the diets were initiated.

### *Diets*

The standard food was rat and mouse breeding diet, which contained 50% carbohydrate and supplied 11.5% of the energy as fat. An isocaloric, isonitrogenous diet supplying 68% of the calories as MCT was calculated based on the composition of the normal diet and was formulated as a paste to minimize food scatter as previously described (Tisdale *et al.*, 1987). The LCT was formulated as a chocolate also to minimize wastage and supplied 69% of the calories as fat derived from cocoa buter and was isonitrogenous with the other diets as calculated by the relevant compositions supplied by Cadbury Schweppes. D-(-)3-hydroxybutyrate was presented as the sodium salt in the drinking water at a concentration of 30 $\mu$mol ml$^{-1}$. Both food and water intake were monitored daily and scattered food was collected in a tray under the cages and subtracted to give the actual food intake. The average daily water consumption per mouse for the groups containing D-(-)3-hydroxybutyrate was 4.1 ml. Body weights were measured daily at the same time of day. At the end of the study blood samples were removed from animals under anaesthesia by cardiac puncture and were collected in heparinized syringes. All blood samples were taken between 9.00 and 11.00 a.m. Blood glucose and FFA were determined immediately and the remaining samples were deproteinized for determination of acetoacetate, 3-hydroxybutyrate and lactate. Results were analysed statistically using the analysis of variance or $F$-ratio.

### *Metabolic assays*

*Blood glucose* Whole blood (0.2 ml) was used and glucose was determined using the *o*-toluidine reagent kit (Sigma). *Acetoacetate and 3-hydroxybutyrate* levels were measured by the method of Mellanby and Williamson (1974) and Williamson and Mellanby (1974) respectively. *Lactate* levels were determined by the method of Gutmann and Wahlefield (1974). FFA levels were measured with a Wako NEFAC kit.

### Results

The effect of dietary modification on weight loss and tumour weight in mice bearing the MAC16 tumour is shown in Figure 1 and Table I. The average daily food consumption by all dietary groups was not significantly different and water consumption also did not vary. The groups containing D-(-)3-hydroxybutyrate in the drinking water consumed ~120 $\mu$mol day$^{-1}$. Animals fed the normal diet lost ~20% of their body weight during the course of the study and this weight loss was only significantly reduced in the animals fed 68% MCT plus 3-hydroxybutyrate ($P$<0.05). Tumour weights appeared to be reduced in all animals fed the high fat diets, although this only reached significance in the groups fed 68% MCT with or without 3-hydroxybutyrate, where the tumour weight was only ~50% of that found in the group consuming normal laboratory pellets ($P$<0.05).

Animals fed the LCT diet had higher levels of circulatory FFA than those fed MCT and normal diets, and this became

**Figure 1** Effect of dietary modification on weight loss in male NMRI mice bearing the MAC16 adenocarcinoma. Dietary groups: A – normal diet; B – 68% MCT; C – 68% MCT+3-hydroxybutyrate; D – 69% LCT. E – 69% LCT+3-hydroxybutyrate. Animals were fed the different diets for the periods indicated in **Materials and methods**.

statistically significant in the group fed 69% LCT+3-hydroxybutyrate ($P$<0.05). Acetoacetate levels were significantly elevated from animals fed the normal diet only in the groups fed MCT. In contrast 3-hydroxybutyrate levels were elevated over the control in all groups except those fed the 69% LCT diet alone. The group fed 68% MCT+3-hydroxybutyrate had significantly higher plasma levels of 3-hydroxybutyrate than the group fed LCT+3-hydroxybutyrate. Blood glucose levels were not significantly altered in any of the dietary groups. Plasma lactate levels were significantly reduced in both the 68% MCT+3-hydroxybutyrate and in the 69% LCT groups.

### Discussion

We have previously shown that both weight loss and tumour weight are reduced in animals bearing the MAC16 tumour when they are fed a ketogenic diet (Tisdale *et al.*, 1987). Ketone bodies might be expected to preserve lean body mass during periods of weight loss and to be poor metabolic substrates for the tumour (Tisdale & Brennan, 1983; Rofe *et al.*, 1986). Additional rationalization for the use of a ketogenic diet for the reversal of the weight loss in cancer cachexia has come from recent studies showing inhibition of lipolytic and proteolytic factors produced by the MAC16 tumour by sodium D-(-)3-hydroxybutyrate (Beck & Tisdale, 1987). Such

**Table I** Effect of dietary modification on weight loss, tumour weight and plasma metabolite levels in NMRI mice bearing the MAC16 adenocarcinoma[a]

| | *Dietry group* | | | | |
|---|---|---|---|---|---|
| *Parameter* | *Normal* | *68% MCT* | *68% MCT+ 3-hydroxybutyrate* | *69% LCT* | *69% LCT+ 3-hydroxybutyrate* |
| Initial weight (g) | 28.5 ±0.5 | 29.3 ±0.9 | 28.3 ±0.4 | 28.6 ±0.5 | 28.7 ±1.1 |
| Final weight (g) (– tumour weight) | 23.6 ±1.9 | 26.8 ±1.2 | 27.0 ±1.2 | 25.7 ±1.2 | 25.5 ±2.1 |
| Weight loss (g) | 4.9 ±1.8 | 2.5 ±1.4 | 1.3 ±1.4$^b$ | 3.0 ±1.6 | 3.2 ±1.4 |
| Food intake (Kcal mouse$^{-1}$ day$^{-1}$) | 15.1 ±0.8 | 14.2 ±1.0 | 14.0 ±0.7 | 16.4 ±1.4 | 15.8 ±0.8 |
| Tumour weight (g) | 0.45 ±0.14 | 0.21 ±0.09$^b$ | 0.19±0.05$^b$ | 0.29 ±0.12 | 0.26 ±0.11 |
| FFA (mM) | 0.39 ±0.11 | 0.49 ±0.11 | 0.50±0.05 | 0.66 ±0.27 | 0.74 ±0.14$^b$ |
| Acetoacetate (mM) | 0.06 ±0.01 | 0.165±0.05$^b$ | 0.18±0.12 | 0.05 ±0.01 | 0.06 ±0.01 |
| 3-Hydroxybutyrate (mM) | 0.064±0.002 | 0.13 ±0.015$^c$ | 0.28±0.03$^c$ | 0.056±0.009 | 0.108±0.022$^b$ |
| Glucose (mg 100 ml$^{-1}$) | 95±13 | 117±20 | 81±7 | 107±10 | 100±9 |
| Lactate (mM) | 13.3 ±1.9 | 11.7 ±0.8 | 9.5 ±0.8$^b$ | 8.8 ±1.1$^b$ | 12.1 ±1.3 |

[a]Results are means±s.e.m.; [b]$P$<0.05 from tumour-bearing group fed a normal diet; [c]$P$<0.005 from tumour-bearing group fed a normal diet.

tumour elaborated catabolic factors are found in the circulation of animals bearing the MAC16 tumour and are thought to be responsible for the cachexia.

To investigate other dietary lipids as anticachectic agents we have compared the ability of MCT and LCT to reduce weight loss and tumour size in the MAC16 cachexia model. In our initial studies we utilized MCT emulsion as a caloric source since the yield of ketone bodies was expected to be higher than for LTC (Cotter *et al.*, 1987). Although ketosis is absent in cancer cachexia, tumour-bearing animals respond to starvation with an enhanced ketonaemia and marked ketonuria, when compared with non tumour-bearing controls (Rofe *et al.*, 1986). This shows that there is no functional impairment of the ketogenic capacity of the liver in the tumour-bearing state, and this has been confirmed by the increased plasma levels of acetoacetate and 3-hydroxybutyrate previously observed in weight-losing, tumour-bearing animals fed high levels of MCT (Tisdale *et al.*, 1987). Therefore an anticachectic diet should give the highest level of ketone bodies achievable for a given level of dietary fat.

Diets containing MCT produced a higher plasma level of both acetoacetate and 3-hydroxybutyrate than a comparable LCT diet. The plasma level of 3-hydroxybutyrate has previously been shown to be elevated after MCT ingestion, but not after LCT ingestion (Seaton *et al.*, 1986; Cotter *et al.*, 1987). Diets containing MCT also protected against weight loss produced by the MAC16 tumour and reduced tumour weight to a greater extent than those containing LCT, even when the latter diets were supplemented with 3-hydroxybutyrate. Some tumours, such as Ehrlich ascites tumour cells, have been shown to metabolize exogenous long chain fatty acid (16 to 18 carbon atoms) rapidly relative to the limited ability to metabolize those of shorter chain length (Spector & Steinberg, 1967). This could pose a potential problem of an enhanced tumour growth in some cases if LCT are used as a source of dietary lipid. In the present study, however, there was no increase in tumour growth in the animals fed the LCT diets.

Another potential problem in feeding patients a high fat diet is the ability of dietary lipids, in particularly unsaturated fat, to promote tumour development and metastasis, particularly with mammary tumours (Sylvester *et al.*, 1986; Abraham & Hillyard, 1983; Katz & Boyulan, 1987). However, using the N-nitrosourea rat mammary tumour model, Cohen and Thompson (1987) have shown that a MCT-containing diet failed to promote tumour development, when compared with a high fat corn oil group, indicating that tumour promotion by dietary fat is more a function of the type than the amount ingested.

Since weight loss produced by the MAC16 tumour is proportional to tumour weight (Bibby *et al.*, 1987; Beck & Tisdale, 1987) it is possible that the prevention of weight loss by the MCT diets is due to a reduction in tumour weight. However, we have previously shown (Tisdale *et al.*, 1987) that a high MCT diet reduces weight loss to a greater extent than might be anticipated from the reduction in tumour size. Moreover, 3-hydroxybutyrate has no effect on the growth of the MAC16 *in vitro* at concentrations up to 6 mM, suggesting no direct antitumour effect.

Although the MAC16 tumour has a large necrotic centre (Bibby *et al.*, 1987) there is no evidence for the involvement of tumour necrosis factor in the production of the cachectic state (Mahony *et al.*, 1988). Tumours from animals fed the MCT diets are possibly less necrotic than those fed a normal diet, but no less necrotic than a tumour of comparable size from animals fed a normal diet. The contribution of a reduction in necrosis to the observed reduction in tumour weight is currently being investigated.

The only potential disadvantage in using MCT is that they have been shown to increase the basal metabolic rate more than LCT (Seaton *et al.*, 1986). This could potentially pose problems in cachectic patients with an already elevated basal metabolic rate (Theologides, 1979), although none have been observed in our initial clinical study (Fearon *et al.*, 1988). Otherwise it is suggested that a high MCT diet supplemented with 3-hydroxybutyrate would be most suitable for clinical studies in cachectic cancer patients.

This work has been supported by a grant from the Cancer Research Campaign. We thank Mr M.P. Wynter for the tumour transplantations.

### References

ABRAHAM, S & HILLYARD, L.A. (1983). Effect of dietary 18-carbon fatty acids on growth of transplantable mammary adenocarcinomas in mice. *J. Natl Cancer Inst.*, **71**, 601.

BECK, S.A. & TISDALE, M.J. (1987). Production of lipolytic and proteolytic factors by a murine tumor producing cachexia in the host. *Cancer Res.*, **22**, 5919.

BIBBY, M.C., DOUBLE, J.A., ALI, S.A., FEARON, K.C.H., BRENNAN, R.A. & TISDALE, M.J. (1987). Characterisation of a cachectic transplantable adenocarcinoma of the mouse colon. *J. Natl Cancer Inst.* **78**, 539.

COHEN, L.A. & THOMPSON, D.O. (1987). The influence of dietary medium chain triglycerides on rat mammary tumor development. *Lipids*, **22**, 455.

COTTER, R., TAYLOR, C.A., JOHNSON, R. & ROWE, W.B. (1987). A metabolic comparison of pure long-chain triglyceride lipid emulsion (LCT) and various medium-chain triglycerides (MCT)–LCT combination emulsions in dogs. *Am. J. Clin. Nutr.*, **45**, 927.

CONYERS, R.A.J., NEED, A.G., DURBRIDGE, T., HARVEY, N.D.M., POTEZNEY, N. & ROFE, A.M. (1979). Cancer ketosis and parental nutrition. *Med. J. Aust.*, **1**, 398.

DE WYS, W.D., BEGG, C. & LAVIN, P.T. (1980). Prognostic effect of weight loss prior to chemotherapy in cancer patients. *Am. J. Med.*, **69**, 491.

DE WYS, W.D. (1986). Weight loss and nutritional abnormalities in cancer patients: Incidence, severity and significance. *Clin. in Oncol.*, **5**, 251.

FEARON, K.C.H., BORLAND, W., PRESTON, T., TISDALE, M.J., SHENKIN, A. & CALMAN, K.C. (1988). Influence of systemic ketosis on substrate levels and nitrogen metabolism in cancer cachexia. *Am. J. Clin. Nutr.* **47**, 42.

GUTMAN, I. & WAHLEFIELD, A.W. (1974). L-(+)-Lactate determination with lactate dehydrogenase and NAD. In *Methods of Enzymatic Analysis*, **4**, Bergmeyer, H.U. (ed) p. 1464. Academic Press: London.

KATZ, E.B. & BOYLAN, E.S. (1987). Stimulatory effect of high polyunsaturated fat diet on lung metastasis from the 13762 mammary adenocarcinoma in female retired breeder rats. *J. Natl Cancer Inst.* **79**, 351.

MAGEE, B.A., POTEZNEY, N., ROFE, A.M. & CONYERS, R.A.J. (1979). The inhibition of malignant cell growth by ketone bodies. *J. Exp. Biol. Med. Sci.*, **57**, 529.

MAHONY, S.M., BECK, S.A. & TISDALE, M.J. (1988). Comparison of weight loss induced by recombinant tumour necrosis factor with that produced by a cachexia-inducing tumour. *Br. J. Cancer*, **57**, 385.

MELLANBY, J. & WILLIAMSON, D. (1974). Acetoacetate. In *Methods of Enzymatic Analysis*, **4**, Bergmeyer, H.U. (ed) Academic Press: London.

ROFE, A.M., BAIS, R. & CONYERS, R.A.J. (1986). Ketone-body metabolism in tumour-bearing rats. *Biochem. J.*, **233**, 485.

SEATON, T.B., WELLE, S.L., WARENKO, M.K. & CAMPBELL, R.G. (1986). Thermic efffect of medium-chain and long-chain triglycerides in man. *Am. J. Clin. Nutr.*, **44**, 630.

SPECTOR & STEINBERG (1967). The effect of fatty acid structure on utilization by Ehrlich ascites tumor cells. *Cancer Res.*, **27**, 1587.

SYLVESTER, P.W., IP, C. & IP, MM. (1986). Effects of high dietary fat on the growth and development of ovarian-independent carcinogen-induced mammary tumours in rats. *Cancer Res.*, **46**, 763.

THEOLOGIDES, A. (1979). Cancer cachexia, *Cancer*, **43**, 2004.

TISDALE, M.J. (1982). Tumour and host nutrition. *Cancer Topics*, **3**, 113.

TISDALE, M.J. & BRENNAN, R.A. (1983). Loss of acetoacetate coenzyme A transferase activity in tumours of peripheral tissues. *Br. J. Cancer*, **47**, 293.

TISDALE, M.J., BRENNAN, R.A. & FEARON, K.C.H. (1987). Reduction of weight loss and tumour size in a cachexia model by a high fat diet. *Br. J. Cancer*, **56**, 39.

WILLIAMSON, D.H. & MELLANBY, J. (1974). D-(-)3-hydroxybutyrate. In *Methods of Enzymatic Analysis*, **4**, Bergmeyer, H.U. (ed) p. 1836. Academic Press: London.

WILLIAMSON, J.F. & MATTHAEI, K. (1981). Cancer induced body wasting. A review of cancer cachexia and a hypothesis concerning the molecular basis of the condition. *Aust. J. Clin. Sci.*, **2**, 158.